# Latest status of non-tuberculous mycobacteria prevalence in Türkiye and the world: Systematic review

**DOI:** 10.5578/tt.20239609

**Published:** 2023-12-07

**Authors:** İmdat KILBAŞ, Meltem UZUN

**Affiliations:** 1 Medical Microbiology Doctorate Program, Institute of Health Sciences, İstanbul University, İstanbul, Türkiye; 2 Department of Medical Microbiology, İstanbul Faculty of Medicine, İstanbul University, İstanbul, Türkiye

## Abstract

**ABSTRACT**

**
Latest status of non-tuberculous mycobacteria prevalence in Türkiye and the world: Systematic review
**

Non-tuberculous mycobacteria (NTM) can cause diseases not only in indivi- duals with compromised immune systems but also in those with normal immune function. This study aimed to compare the prevalence of NTM in Türkiye and worldwide between 2012 and 2022. This study was designed following the guidelines outlined in the Preferred Reporting Items for Systematic Reviews and Meta-Analyses (PRISMA) procedure. A systematic search was conducted between January 2012 and September 2022 using different electronic databases, including Pubmed, Medline, Embase, Web of Science, Ebsco, Scopus, Türk Medline, and Google Scholar. During the litera- ture review process, titles and abstracts were examined and the full texts of the studies were accessed. In 13 research articles from Türkiye included in the study, a total of 17.293 samples were studied and a total of 1304 NTM (7.54%) strains were isolated from these samples. Among the 1304 NTM strains reported from Türkiye, the top three most frequently isolated species were M. abscessus (29.83%), M. lentiflavum (14.97%), M. fortuitum (14.38%). In 35 studies included from around the world, a total of 512.626 samples were studied and a total of 12.631 NTM (2.46%) strains were isola- ted from these samples. Among the 12631 NTM strains isolated, the top three most frequently isolated species were M. intracellulare (28.13%), M. avium (17.70%) and M. abscessus (14.88%). This study unveiled the global preva- lence of NTM-infected patients, detailing species distribution and microbiolo- gical diagnostic methods. Variations in NTM spread were observed, influen- ced by diverse factors.

**Key words:** Non-tuberculous mycobacteria; prevalence; Mycobacterium abs- cessus; Mycobacterium avium

**ÖZ**

**
Türkiye ve dünyada tüberküloz dışı mikobakteri prevalansında son durum: Sistematik derleme
**

Tüberküloz dışı mikobakteriler (TDM), özellikle immün sistemi baskılanmış bireylerde hastalıklara sebep olmakla beraber, bağışıklık sistemi normal kişiler- de de hastalık oluşturmaktadır. Bu çalışmanın amacı 2012-2022 yılları arasın-

da Türkiye ve dünyadaki TDM prevalansının karşılaştırılmasıdır. Bu çalışma, Sistematik Derlemeler ve Meta Analizler için Tercih Edilen Raporlama Ögeleri (PRISMA) prosedürü kuralları baz alınarak planlanmıştır. Ocak 2012-Eylül 2022 tarihleri arasında Pubmed, Medline, Embase, Web of Science, Ebsco, Scopus, Türk Medline ve Google Scholar dahil olmak üzere farklı elektronik veri tabanları kullanarak sistematik bir tarama gerçekleştirilmiştir. Literatür tarama sürecinde başlık ve özetler incelenmiş ve çalışmaların tam metin- lerine ulaşılmıştır. Türkiye’den çalışmaya dahil edilen 13 araştırma makalesinde toplam 17,293 örnek ile çalışılmış ve bu örneklerden toplam 1304 TDM (%7,54) suşu izole edilmiştir. Türkiye’den bildirilen 1304 TDM suşu içinde en sık izole edilen ilk üç tür sırasıyla;

M. abscessus (%29,83), M. lentiflavum (%14,97), M. fortuitum (%14,38) olarak saptanmıştır. Dünya genelinden dahil edilen 35 çalışmada toplam 512,626 örnekle çalışılmış ve bu örneklerden toplam 12631 TDM (%2,46) suşu izole edilmiştir. İzole edilen 12,631 TDM suşu içinde en sık izole edilen ilk üç türün sırasıyla; M. intracellulare (%28,13), M. avium (%17,70), M. abscessus (%14,88) olduğu saptanmıştır. Bu çalışma sonucunda, dünya çapında TDM’ler ile enfekte hastaların prevalansı, türlerine göre dağılım ve mik- robiyolojik tanı yöntemleri gözler önüne sermiş olup, TDM’lerin yayılımının pek çok farklı faktöre bağlı olarak değiştiği görülmüştür.

**Anahtar kelimeler:** Tüberküloz dışı mikobakteriler; prevalans; mycobacterium abscessus; mycobacterium avium

## INTRODUCTION

As of today, over 160 non-tuberculous mycobacteria (NTM) species have been reported in various natural environments, predominantly found in soil and water resources, including drinking water systems (1). Non- tuberculous mycobacteria (NTM) can cause diseases in individuals with both suppressed and normal immune function. They have the potential to infect multiple systems, primarily the respiratory tract, leading to diverse clinical presentations (2).

The prevalence of NTM varies across countries but has seen a global increase, emerging as a significant public health concern, particularly among the elderly population. Although the exact cause for this increase remains unknown, potential factors include a rise in the patient population susceptible to these bacteria, the advent of advanced molecular-based tests, an increase in environmental sources of infection, and heightened awareness among clinicians regarding NTM (3,4).

Until recently, *Mycobacterium* species were identified by traditional methods based on biochemical/ morphological characteristics, reproduction rate, and other phenotypic tests (5). However, definitions based on these phenotypic features are time-consuming and can also lead to erroneous results (6). Today, molecular methods including polymerase chain reaction (PCR)- based hybridization and sequencing are routinely used in advanced laboratories. These tests have become the new standard tests for identifying mycobacteria. However, although these methods help to quickly identify *Mycobacterium* species, they are quite costly and require a high level of technical knowledge/expertise (7).

Given the global rise, this systematic review was conducted to compare the prevalence of NTM in both Türkiye and worldwide from 2012 to 2022. Our

study will shed light on which species are isolated most frequently, which methods and gene regions are most frequently used in identification, and whether NTM identification methods affect prevalence.

## MATERIALS and METHODS

**Protocol**

The study adhered to the guidelines outlined in the Preferred Reporting Items for Systematic Reviews and Meta-Analyses (PRISMA) procedure (8). The PROSPERO registration number of the study is CRD42022371964.

## Literature Search

A systematic search was conducted between January 2012 and September 2022 using different electronic databases, including Pubmed, Medline, Embase, Web of Science, Ebsco, Scopus, Turkish Medline, and Google Scholar; screening in English and Turkish languages “non-*M. tuberculosis*”, “nontuberculous *Mycobacteria*” “non-* Mycobacterium tuberculosis* in Turkey”, “NTM”, “Tüberküloz dışı mikobakteriler”, “*M. tuberculosis* dışı mikobakteriler”, “TDM”, “*Mycobacteria* identification in Turkey”, “identification of *Mycobacterium* species in Turkey”.

Two independent authors scanned the keywords, titles, and abstracts for relevance. After thorough evaluation, original articles meeting the inclusion criteria had their data included in the study. Out of the total 6518 studies found using the determined keywords in the databases, the full text of 3761 studies was accessed. Following the exclusion criteria, a total of 48 studies were included in the analysis, comprising 35 original research articles from various global locations and 13 from Türkiye.

Studies published in national and international peer- reviewed journals, whose full text can be accessed in

Latest situation in non-tuberculous mycobacteria prevalence

databases, defined at the species level, with more than 20 samples, published in Turkish or English between January 2012 and September 2022 were included in this systematic review.

## Exclusion Criteria

Those whose full text could not be accessed, that were not identified at the species level, that involved animal experiments, that had fewer than 20 samples, that were compilations, systematic reviews, case reports, book chapters, letters to the editor and meta- analyses, that were repetitive, investigated only a single species, had inconsistencies in their data, studies conducted with strains isolated from water

and the environment, published before 2012 and published in languages other than Turkish and English were excluded.

## Evaluation and Analysis of Literature Data

During the literature review process, titles and abstracts were examined and the full texts of the studies were accessed. Microsoft Excel spreadsheets were prepared to collect data, and in these tables, the surname of the first author, year of publication, location of the study, sample size, and information about the identification methods used in the studies were included. Analyses of the data were made using IBM SPSS Statistics for Mac, Version 25.0.

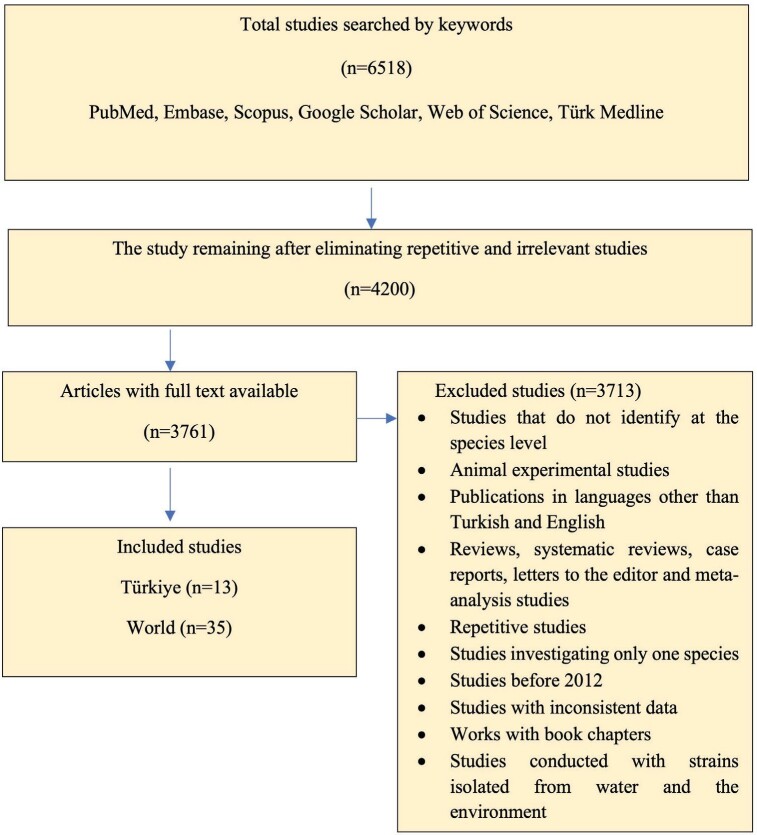

**Figure 1.** Publications excluded within the scope of the search criteria and included in the study (PRISMA flow chart).

## RESULTS

As a result of the literature review, a total of 6518 studies published between 2012 and 2022 were identified. Based on the evaluation of these studies against the inclusion and exclusion criteria, it was found that 13 research articles from Türkiye and 35 research articles from various countries worldwide fulfilled the systematic review criteria (Figure 1). The included publications underwent analysis and were categorized into two periods: 2012-2016 and 2017- 2022.

A total of 17.293 samples were studied in 13 research articles from Türkiye included in the study, where a total of 1304 NTM (7.54%) strains were isolated from these samples. Among the 1304 NTM strains reported from Türkiye, the top five most frequently isolated species were *M. abscessus* (29.83%), *M. lentiflavum* (14.97%), *M. fortuitum* (14.38%), *M. gordonae* (12.66%) and *M. intracellulare* (12.13%). It was determined that the most frequently studied gene regions were hsp65 (eight studies) and 16S rRNA (four studies), and NTMs were most frequently isolated from sputum and BAL, respectively. Among these studies, PCR-based methods along with sequencing were commonly utilized for identification, as observed in five of the research articles. While mean ages were provided in certain studies included in the analysis, it was noted that in others, age- related data were absent. The specifics and characteristics of the studies included from Türkiye are given in Table 1.

A total of 512.626 samples studied in 35 studies from around the world were included in the systematic review, where a total of 12.631 NTM (2.46%) strains were isolated from these samples. Among the 12.631 NTM strains isolated, the top five most frequently isolated species were *M. intracellulare* (28.13%), *M. avium* (17.70%), *M. abscessus* (14.88%), *M. fortuitum* (11.63%) and *M. simiae* (11.29%). In studies conducted around the world, the most frequently studied gene regions were hsp65 (12 studies) and 16S rRNA (11 studies). In these studies, it was observed that PCR-based methods were frequently used for identification (in 17 studies). The strains were most frequently isolated from sputum

and BAL samples. However, it was observed that age- related data were not reported in the majority of studies. The detailed characteristics of the studies included from around the world are presented in Table 2, categorizing all studies into two groups: 2012-2016 and 2017-2022.

It was determined that the prevalence of NTM in Türkiye increased between 2017-2022 compared to 2012-2016 (p> 0.05). In Türkiye, the prevalence of

*M. abscessus, M. gordonae,* and *M. avium* species increased over the years, while the prevalence of

*M. kansasii* and *M. lentiflavum* decreased over the years (p> 0.05). It was determined that the prevalence of NTM in the world increased between 2017-2022 compared to 2012-2016 (p> 0.05). Accordingly, it was observed that the prevalence of *M. gordonae,
*

*M. chelonae,* and *M. simiae* increased over the years, while the prevalence of *M. lentiflavum* and

*M. porcinum* decreased. It was determined that the prevalence of *M. fortuitum* did not change in general (p> 0.05) (Table 3). As certain studies were exclusively designed to focus on NTM strains and their identification, the prevalence might appear as 100% when assessed against the total number of samples in those particular studies. Consequently, eight studies worldwide and three studies in Türkiye were excluded from the calculation of the overall NTM prevalence.

## DISCUSSION

Currently, with the rise in AIDS cases and the implementation of immunosuppressive treatments, infections attributed to NTMs are regarded as a significant contributor to mortality and/or morbidity in lung diseases. The distribution of causative agents varies across different regions (55). In our country, the available data indicates a relatively limited number of studies focused on the distribution of NTM infections.

In recent years, NTMs have emerged as significant pathogens posing a threat to human health. Their incidence and prevalence continue to rise globally (56). Previously regarded solely as contaminants, it has been established over several years that NTMs cause pulmonary and extrapulmonary infections in both immunocompetent and immunosuppressed individuals (42).

**Table:** 

**Table 1.** Studies and data included from Turkish literature																	
**Study name**	**Gene region**	**Method**	**Total sample**	**NTM (+)**	***M. M.*** ***fortuitum, abscessus,*** **n (%) n (%)**		***M.*** ***gordonae,*** **n (%)**	***M.*** ***avium,*** **n (%)**	***che***	***M. M.*** *** lonae, intracellulare, *** **n (%) n (%)**			***M.*** ***kansasii,*** **n (%)**	***M.*** ***lentiflavum,*** **n (%)**	***M.*** ***porcinum,*** **n (%)**	***M.*** ***simiae,*** **n (%)**	**Other NTM’s,** **n (%)**
Albayrak et al. (9)	-	MGIT 960. CM/AS	2491	75	25 (33.33)	14 (18.66)	8 (10.66)	6 (8)		3 (4)		3 (4)	3 (4)	-	-	-	13 (17.33)
Kılıçaslan et al. (10)	hsp65	PCR-REA Method	42	40	3 (7.5)	14 (35)	-	9 (22.5)				2 (5)	8 (20)	-	-		4 (10)
Babalık et al. (11)	hsp65	PCR	75	26	5 (19.23)	9 (34.61)	3 (11.53)	-		-		-	5 (19.23)		-	-	4 (15.38)
Gunaydın et al. (12)	16S rRNA and hsp65	Sequencing	90	90	8 (8.88)	13 (14.44)	21 (23.33)		1	(1.11)	6	(6.66)		9 (10)	-	-	32 (35.55)
Satana et al. (13)	-	Bactec 460 TB. CM/AS	28	23		14 (68.86)	-	-		-		-	-	9 (39.13)	-	-	-
Özçolpan et al. (14)	hsp65 and 16S rDNA	NAP. BD MGIT TBC Identificaiton	5122	126	3 (2.38)	6 (4.76)	4 (3.17)	-	2	(1.58)		-	1 (0.79)	36 (28.57)	40 (31.74)	-	34 (26.98)
Samlı and İlki (15)	-	MALDI-TOF MS. MPT 64	69	13	-	1 (7.69)	-	1 (7.69)			5	(38.46)		-	-	-	6 (46.15)
Akyar et al. (5)	hsp65 and 16S rRNA	MALDI-TOF MS. Sequencing	155	95	6 (6.31)	11 (11.57)	18 (18.94)	4 (4.21)	7	(7.36)	12	(12.63)	8 (8.42)	4 (4.21)	-	6 (6.31)	19 (20)
Appak et al. (3)	hsp65	PCR. Sequencing. MGIT 960	150	72	-	69 (95.83)	-	1 (1.38)		-		-	-	-	1 (1.38)	-	1 (1.38)
Ceyhan et al. (16)	23S rRNA	PCR	435	435	-	-	-	77 (17.70)		-	70	(16.09)	10 (2.29)	-	-	9 (2.06)	269 (61.83)
Erkose Genc et al. (17)	hsp65	MALDI-TOF MS. PCR. Sequencing	152	152	28 (18.42)	66 (43.42)	3 (1.97)	16 (10.52)	4	(2.63)		-	4 (2.63)	8 (5.26)	1 (0.65)	5 (3.28)	17 (11.18)
Özen et al. (18)	hsp65 and 16S rRNA	Sequencing	1004	112	10 (8.92)	15 (13.39)	28 (25)	13 (11.60)	8	(7.14)	6	(5.35)	8 (7.14)	3 (2.67)	3 (2.67)	6 (5.35)	12 (10.71)
Sumbul et al. (19)	-	LJ ve MGIT 960	7480	45	11 (24.44)	8 (17.77)	3 (6.66)	2 (4.44)	4	(8.88)	4	(8.88)	2 (4.44)	-	-	5 (11.11)	6 (13.33)

**Table:** 

**Table 2.** Studies and data included from global literature
***M. M. M. M. M. M. M. M. M. M.* Other Total NTM *fortuitum*, *abscessus*, *gordonae*, *avium*, *intracellulare*, *chelonae*, *kansasii*, *lentiflavum*, *porcinum*, *simiae*, NTM’s, ** ** Study name Country Gene region Method sample (+) n (%) n (%) n (%) n (%) n (%) n (%) n (%) n (%) n (%) n (%) n (%) **
Christianson Canada hsp65 PCR 115 155 - 10 (6.45) 46 10 (6.45) - - - 50 (32.25) et al. (20) (29.67)
Park et al. Korea NA HPLC and 133 133 6 (4.51) 16 2 (1.50) 17 39 (29.32) - 48 - - - 5 (3.75) (21) PCR (12.03) (12.78) (36.09)
Lima et al. Brazil hsp65 PCR 1812 75 4 (5.33) 24 (32) 1 (1.33) 13 1 (1.33) - - - 1 (1.33) 1 13 (17.33) (22) (17.33) (1.33)
Balada- USA 16S rRNA MALDI-TOF 178 142 16 23 15 - - 24 (16.45) 14 1 (0.70) - - 49 (34.50) Llasat et al. hsp65 MS. PCR (11.26) (16.19) (10.56) (9.85) (23)
Singh et al. India 23S rRNA CM/AS 1080 60 20 8 (13.33) 1 (1.66) - 11 (18.33) 4 (6.66) 2 (3.33) - - 1 13 (21.66) (24) (33.33) (1.66)
Yu et al. (25) China 16S rRNA. Sequencing 3995 91 8 (8.79) 33 3 33 (36.26) 2 (2.19) - 3 (3.29) - 9 (9.89) 16S-23S (36.26) (3.29) rRNA
Zwaan et al. Holland rpoB. 16S Sequencing 455 384 - 38 (9.89) 62 112 18 (4.68) 21 (5.46) 40 - - 5 88 (22.91) (26) rRNA (16.14) (29.16) (10.41) (1.30)
Tudó et al. Spain NA MALDI-TOF 243 180 8 (4.44) 31 6 (3.33) 53 30 (16.66) 6 (3.33) 13 - - 1 28 (15.55) (27) MS (17.22) (29.44) (7.22) (0.55)
Maurya et India NA CM/AS 756 62 17 9 (14.51) 2 (3.22) 13 (20.96) 8 (12.90) 3 (4.83) - - - 10 (16.12) al. (28) (24.41)
Mwikuma et Zambia 16S-23S Sequencing 91 54 4 (7.40) - 4 (7.40) 8 15 (27.77) - - 9 (16.66) - - 14 (25.92) al. (29) rRNA (14.81)
Wang et al. Korea rpoB PCR 279 84 5 (5.95) - 3 (3.57) 18 38 (45.23) 1 (1.19) 2 (2.38) - - 12 (14.28) (30) (21.42)
Mediavilla- Spain hsp65 MALDI-TOF 65 65 6 (9.23) 4 (6.15) 4 (6.15) 16 13 (20) 5 (7.69) 4 (6.15) 3 (4.61) 3 (4.61) - 7 (10.76) Gradolph et MS. CM/AS. (24.61) al. (31) PCR
Khosravi et Iran rpoBC PCR 107 88 20 - - 5 1 (1.13) 1 (1.13) 10 - - 41 11 (12.5) al. (32) (22.72) (5.68) (11.36) (46.59)
Otchere et Ghana IS6110. PCR 2036 89 5 (5.61) - 43 18 (20.22) - - - - 1 13 (14.60) al. (33) hsp65. (48.31) (1.12)
Nasiri et al. Iran 16S rRNA. Sequencing 7600 62 10 3 (4.83) 2 (3.22) - 5 (8.06) - 11 - - 21 4 (6.45) (34) rpoB. hsp65 (16.12) (17.74) (33.87)
Verma et al. India Hsp 65 PCR 121 121 42 11 (9.09) - - - 57 (47.10) - - - 11 (9.09) (35) (34.70)
Tan et al. China 16S Sequencing 607 607 17 (2.80) 246 6 (0.98) 99 171 (28.17) 61 - - - 7 (1.15) (36) rRNA. hsp65. (40.52) (16.30) (10.04) rpoB. 16S– 23S rRNA
Gharbi et al. Tunisia rpoB. 16S Sequencing 10.466 30 5 (16.66) - - - - 3 (10) 7 - 1 (3.33) - 14 (46.66) (37) rRNA. hsp65. (23.33) sodA

**Table:** 

**Table 2.** Studies and data included from global literature (continue)
***M. M. M. M. M. M. M. M. M. M.* Other Total NTM *fortuitum*, *abscessus*, *gordonae*, *avium*, *intracellulare*, *chelonae*, *kansasii*, *lentiflavum*, *porcinum*, *simiae*, NTM’s, ** ** Study name Country Gene region Method sample (+) n (%) n (%) n (%) n (%) n (%) n (%) n (%) n (%) n (%) n (%) n (%) **
Furuuchia. Japan NA MGIT 960. 18298 2155 28 (1.29) 119 - 1881 9 (0.41) 83 7 (0.32) 2 (0.09) 3 23 (1.06) et al. (38) PCR (5.52) (87.28) (3.85) (0.13)
Lin et al. China 16S rRNA. Sequencing 1425 60 2 (3.33) 6 (10) - 4 41 (68.33) 4 (6.66) - - 3 (5) (39) rpoB. (6.66) 16S rRNA
Pan et al. China Rv0577. PCR 183 122 - 9 (7.37) - 2 25 (20.49) - - 5 (4.04) - - 72 (59.01) (40) Hsp65 (1.63)
Zarandi et Iran 16s rRNA LPA. PCR 5061 42 12 3 (7.14) - 1 1 (2.38) 7 - - 17 - al. (41) (28.57) (2.38) (16.66) (40.47)
Sharma et India 16S rRNA MGIT 960 2.938 35 12 4 (11.42) - 2 (5.71) 4 (11.42) 2 (5.71) - - 1 10 (28.57) al. (42) (34.28) (2.85)
Sam et al. India 16S rRNA Sequencing. 81777 170 10 (5.88) 26 - 6 22 (12.94) - 2 (1.17) - - 8 96 (54.47) (43) MALDI-TOF (15.29) (3.52) (4.70)
Singh et al. India NA PCR 50 50 - 20 (40) - 4 (8) - 26 (52) - - - - (44)
Huang et al. China NA DNA 1514 223 8 (3.58) 48 - 16 71 (31.83) - 5 (2.24) - - - 16 (7.17) (45) microarray- (21.52) (7.17) chip
Smirnova et Russia NA PCR 247 210 15 (7.14) 22 20 (9.52) 25 27 (12.85) 21 (10) 22 25 (11.90) - 1 29 (13.80) al. (46) (10.47) (11.90) (10.47) (0.47)
Lee et al. Korea NA CM/AS 345.871 4988 266 614 - 33 2762 (55.37) 50 (1) 151 39 (0.78) - - 104 (2.08) (47) (5.33) (12.30) (0.66) (3.02)
Huang et al. China 16S rDNA PCR 308 218 - 27 - 48 131 (60.09) 5 (2.29) - - - 7 (9.85) (48) (12.38) (22.01)
Asaoka et Japan NA BACTEC 213 213 27 30 69 - - 22 (10.32) 42 - - 2 21 (9.85) al. (49) MGIT 960 (12.67) (14.08) (32.39) (19.71) (0.93)
Kim et al. Korea NA PA. PCR 320 320 9 (2.81) 2 (0.62) 4 (1.25) 29 234 (73.12) - 4 (1.25) - - - 18 (5.62) (50) (9.06)
Huang et al. China NA PCR 837 22 - 1 (4.54) - 4 15 (68.18) - 1 (4.54) - - - 1 (4.54) (51) (18.18)
Almutairi et Saudi NA PCR 183 95 5 (5.26) 5 (5.26) 14 5 - 5 (5.26) 2 (2.10) - - 21 38 (40) al. (52) (14.73) (5.26) (22.10)
Song et al. Korea NA MALDI-TOF 124 124 5 (4.03) 53 3 (2.41) 35 22 (17.74) - 5 (4.03) - - - 1 (1.80) (53) MS (42.74) (28.22)
Zhu et al. China 16S rRNA. PCR 23138 1102 9 (0.81) 182 36 (3.26) 145 604 (54.80) 182 90 - - - 36 (3.26) (54) hsp65. (16.51) (13.15) (16.51) (8.16)
CM/AS: GenoType® *mycobacterium* CM/AS assay, PA: Phylogenetic analyses, HB: Hybridization, Saudi: Saudi Arabia, LPA: Line probe assay.

**Table:** 

**Table 3.** Prevalence of non-tuberculous mycobacteria in Türkiye and the world						
		**Türkiye**				**World**
	**Year**		**n**	**Mean (%) ± SS**	**n**	**Average (%) ± SS**
	2012-2016		7	23.62 ± 31.53	12	16.72 ± 28.54
NTM prevalance						
	2017-2022		6	53.92 ± 34.76	23	40.26 ± 37.41
	2012-2016		7	10.19 ± 12.21	10	11.77 ± 10.14
*M. fortuitum*						
	2017-2022		6	9.68 ± 9.93	18	11.56 ± 11.39
	2012-2016		7	25.15 ± 19.74	12	13.67 ± 11.14
*M. abscessus*						
	2017-2022		6	30.33 ± 35.15	23	12.92 ± 12.36
	2012-2016		7	6.95 ± 8.77	10	5.49 ± 4.77
*M. gordonae*						
	2017-2022		6	8.76 ± 10.69	8	8.48 ± 10.76
	2012-2016		7	5.45 ± 8.37	12	15.21 ± 11.98
*M. avium*						
	2017-2022		6	8.31 ± 6.06	23	12.84 ± 19.85
	2012-2016		7	7.73 ± 13.82	12	5.05 ± 5.50
*M. intracellulare*						
	2017-2022		6	7.16 ± 6.61	23	5.02 ± 10.41
	2012-2016		7	0.95 ± 1.49	10	22.06 ± 13.35
*M. chelonae*						
	2017-2022		6	4.33 ± 3.95	16	33.69 ± 25.25
	2012-2016		7	6.28 ± 9.21	9	9.17 ± 10.53
*M. kansasii*						
	2017-2022		6	4.15 ± 3.16	20	10.32 ± 11.83
	2012-2016		3	25.9 ± 14.74	3	7.33 ± 8.32
*M. lentiflavum*						
	2017-2022		3	4.05 ± 1.3	4	4.28 ± 5.35
	2012-2016		1	31.75	3	3.08 ± 1.656
*M. porcinum*						
	2017-2022		3	1.58 ± 1.0	2	1.71 ± 2.291
	2012-2016		0	-	4	1.21 ± 0.468
*M. simiae*						
	2017-2022		5	5.63 ± 3.491	10	15.33 ± 18.64
	2012-2016		7	21.63 ± 15.72	12	18.75 ± 9.11
Other NTM’s (%)						
	2017-2022		6	19.74 ± 21.47	23	14.39 ± 18.39
n: Number of studies.

When the results of the studies included in this systematic review were examined, it was seen that there were differences in species distribution. The top three species most frequently isolated in studies reported from Türkiye were *M. abscessus* (29.83%), *M. lentiflavum* (14.97%), *M. fortuitum* (14.38%). In studies published around the world, the top three most frequently isolated species were *M. intracellulare* (28.13* %), M. avium* (17.70%), *M. abscessus* (14.88%). Zulu et al. (2021) stated that the most frequently isolated NTM species from humans were *M. intracellulare, M. lentiflavum,* and *M. avium*, respectively, in their systematic review (57). In the systematic review published by Bachar et al. (2022), the most frequently isolated species included members of the *M. avium* complex (65%) (58). In the systematic review of Dahl et al. (2022), the most frequently isolated species included members of the *M. abscessus*

complex (83.9%) (59). In another study conducted in Taiwan, the most frequently isolated mycobacteria included the *M. abscessus* complex and intra-complex members (60). These differences were attributed to the sensitivity of the patient population included in the studies to NTMs, the distribution of the immunocompromised patient population by country and region, the socioeconomic status and geographical conditions of the countries, and the difference in the environmental distribution of mycobacteria.

Upon reviewing the findings of the studies included in the compilation, it’s evident that there are regional disparities not only globally but also within Türkiye. Nevertheless, it is noteworthy that species such as *M. intracellulare* and *M. avium*, commonly isolated from AIDS patients, occupied the top two positions. Hence, it has been observed that the distribution of

NTMs is intricately linked to both the diseases present and the host’s immune system.

It has been noted that the clinical samples from which NTMs are most frequently isolated for identification have remained consistent, both within our country and globally. Primarily, sputum and BAL samples continue to be the most commonly utilized, respectively.

In the studies included in this systematic review, it was determined that the most frequently studied gene regions in Türkiye were hsp65 (eight studies) and 16S rRNA (four studies), while in studies conducted worldwide, they were hsp65 (12 studies) and 16S rRNA (11 studies).

## CONCLUSION and RECOMMENDATIONS

This systematic review aimed to compare the prevalence of NTM in Türkiye and the world between 2012 and 2022. The findings revealed that the dissemination of NTM varies significantly due to numerous factors.

The findings of our study revealed data on the prevalence of patients infected with NTMs worldwide, distribution by species, and microbiological diagnostic methods. However, it was observed that the majority of the studies included in the systematic review did not include data such as drug resistance/ susceptibility results of the strains, detailed sociodemographic information of the patients, and factors that predispose them to infection. Incorporating information on drug resistance, comorbidities, and diverse clinical characteristics of patients in future publications detailing NTM infections will aid in devising novel strategies for diagnosing, treating, and preventing these infections. Additionally, such comprehensive data will promote the adoption of the ‘One Health’ approach.

## CONFLICT of INTEREST

The authors declare that they have no conflict of interest.

## AUTHORSHIP CONTRIBUTIONS

Concept/Design: MU, İK Analysis/Interpretation: MU, İK Data acqusition: MU, İK Writing: MU, İK

Clinical Revision: MU, İK Final Approval: MU, İK

## REFERENCES

1. *
  Bai A, Belda O, Dosanjh A. Pulmonary nontuberculous mycobacterial infection in infants: A systematic review. Pediatric Health Med Ther 2021; 12: 551-9. https://doi. org/10.2147/PHMT.S332434*
2. *
  Guglielmetti L, Mougari F, Lopes A, Raskine L, Cambau E. Human infections due to non tuberculous mycobacteria: The infectious diseases and clinical microbiology special- ists’ point of view. Future Microbiol 2015; 10(9): 1467- 83. https://doi.org/10.2217/fmb.15.64*
3. *
  Appak Ö, Türkel S, Esen N, Özkütük AA. Comparison of polymerase chain reaction-restriction enzyme analysis method and DNA sequence analysis results in the identi- fication of non-tuberculous mycobacteria. Acta Microbiol Immunol Hung 2018; 65(4): 515-27. https://doi. org/10.1556/030.65.2018.027*
4. *
  Jarzembowski JA, Michael B, Young MD. Non tuberculous mycobacterial infections. Arch Pathol Lab Med 2008; 132(8): 1333-41. https://doi.org/10.5858/2008-132- 1333-NMI*
5. *
  Akyar I, Çavuşoğlu C, Ayaş M, Sürücüoğlu S, İlki A, Kaya DE, et al. Evaluation of the performance of MALDI-TOF MS and DNA sequence analysis in the identification of mycobacteria species. Turk J Med Sci 2018; 48(6): 1351- 7. https://doi.org/10.3906/sag-1801-198*
6. *
  El Khéchine A, Couderc C, Flaudrops C, Raoult D, Drancourt M. Matrix-assisted laser desorption/ionization time-of-flight mass spectrometry identification of myco- bacteria in routine clinical practice. PLoS One 2011; 6(9): e24720. https://doi.org/10.1371/journal. pone.0024720*
7. *
  Leyer C, Gregorowicz G, Mougari F, Raskine L, Cambau E, de Briel D. Comparison of Saramis 4.12 and IVD 3.0 Vitek MS matrix-assisted laser desorption ionization-time of flight mass spectrometry for identification of mycobacte- ria from solid and liquid culture media. J Clin Microbiol 2017; 55(7): 2045-54. https://doi.org/10.1128/ JCM.00006-17*
8. *
  Page MJ, McKenzie JE, Bossuyt PM, Boutron I, Hoffmann TC, Mulrow CD, et al. The PRISMA 2020 statement: An updated guideline for reporting systematic reviews. BMJ 2021; 372: 71. https://doi.org/10.1136/bmj.n71*
9. *
  Albayrak N, Simşek H, Sezen F, Arslantürk A, Tarhan G, Ceyhan I. Ulusal Tüberküloz Referans Laboratuvarında 2009-2010 yıllarında tespit edilen tüberküloz dışı miko- bakterilerin dağılımlarının irdelenmesi [Evaluation of the distribution of non-tuberculous mycobacteria strains iso- lated in National Tuberculosis Reference Laboratory in 2009-2010, Turkey]. Mikrobiyol Bul 2012; 46(4): 560-7.
  *
10. *
  Kılıçaslan Z, Kuyucu T, Babalık A, Özkan G, Kaya Bingöl Z, Ordu EN, et al. Akciğerde tüberküloz dışı mikobakteri hastalığı: 42 vakanın retrospektif analizi. J Med Sci 2013; 33(3): 854-67. https://doi.org/10.5336/meds- ci.2012-32048*
11. *
  Babalık A, Kuyucu T, Ordu EN, Ernam D, Partal M, Köksalan K. Non-tuberculous mycobacteria infection: 75 cases. Tuberk Toraks 2012; 60(1): 20-31. https://doi. org/10.5578/tt.2543*
12. *
  Gunaydin M, Yanik K, Eroglu C, Sanic A, Ceyhan I, Erturan Z, et al. Distribution of nontuberculous Mycobacteria strains. Ann Clin Microbiol Antimicrob 2013;12:33. doi: 10.1186/1476-0711-12-33.* *https://doi.* *org/10.1186/1476-0711-12-33*
13. *
  Satana D, Erkose-Genc G, Tamay Z, Uzun M, Guler N, Erturan Z. Prevalence and drug resistance of mycobacteria in Turkish cystic fibrosis patients. Ann Clin Microbiol Antimicrob 2014; 13: 28. https://doi.org/10.1186/1476- 0711-13-28*
14. *
  Özçolpan OO, Sürücüoğlu S, Özkütük N, Çavuşoğlu C. Klinik örneklerden soyutlanan ve DNA dizi analizi ile tanımlanan tüberküloz dışı mikobakterilerin dağılımı [Distribution of nontuberculous mycobacteria isolated from clinical specimens and identified with DNA sequence analysis]. Mikrobiyol Bul 2015; 49(4): 484-93. https://doi. org/10.5578/mb.9698*
15. *
  Şamlı A, İlki A. Comparison of MALDI-TOF MS, nucleic acid hybridization and the MPT64 immunochromato- graphic test for the identification of M. tuberculosis and non-tuberculosis Mycobacterium species. New Microbiol 2016; 39(4): 259-63.
  *
16. *
  Ceyhan İ, Özkara Ş, Güler MZ, Dulkar G, Altınsoy R, Vezir
  *

S. Türkiye’de bir referans hastanede akciğer örneklerinden sıklıkla izole edilen yavaş üreyen tüberküloz dışı mikobak- teriler ve ilaç duyarlılık sonuçlarının değerlendirilmesi [frequently isolated slow growing nontuberculous myco- bacteria from pulmonary samples and evaluation of drug susceptibility testing results in a referral hospital in Turkey]. Mikrobiyol Bul 2019; 53(3): 330-5. https://doi. org/10.5578/mb.68091

17. *
  Erkose Genc G, Demir M, Yaman G, Kayar B, Koksal F, Satana D. Evaluation of MALDI-TOF MS for identification of nontuberculous mycobacteria isolated from clinical specimens in mycobacteria growth indicator tube medi- um. New Microbiol 2018; 41(3): 214-9.
  *
18. *
  Özen N, Kula Atik T, Çetin Duran A. Klinik örneklerden izole edilen Mycobacterium tuberculosis kültür ve ilaç duyarlılık test sonuçlarının analizi ve tüberküloz dışı miko- bakterilerin dağılımının incelenmesi [Evaluation of Mycobacterium tuberculosis culture and drug susceptibil- ity test results and the distribution of nontuberculosis mycobacteria from the clinical specimens]. Mikrobiyol Bul 2020; 54(4): 559-74. https://doi.org/10.5578/mb.70088*
19. *
  Sumbul B, Doymaz MZ. A current microbiological picture of Mycobacterium isolates from Istanbul, Turkey. Pol J Microbiol 2020; 69(2): 1-7. https://doi.org/10.33073/ pjm-2020-021*
20. *
  Christianson S, Wolfe J, Soualhine H, Sharma MK. Comparison of repetitive-sequence-based polymerase chain reaction with random amplified polymorphic DNA analysis for rapid genotyping of nontuberculosis myco- bacteria. Can J Microbiol 2012; 58(8): 953-64. https:// doi.org/10.1139/w2012-068*
21. *
  Park JS, Choi JI, Lim JH, Ahn JJ, Jegal Y, Seo KW, et al. The combination of real-time PCR and HPLC for the identifica- tion of non-tuberculous mycobacteria. Ann Lab Med 2013; 33(5): 349-52. https://doi.org/10.3343/ alm.2013.33.5.349*
22. *
  Lima CA, Gomes HM, Oelemann MA, Ramos JP, Caldas PC, Campos CE, et al. Nontuberculous mycobacteria in respiratory samples from patients with pulmonary tuber- culosis in the state of Rondônia, Brazil. Mem Inst Oswaldo Cruz 2013; 108(4): 457-62. https://doi.org/10.1590/ S0074-0276108042013010*
23. *
  Balada-Llasat JM, Kamboj K, Pancholi P. Identification of mycobacteria from solid and liquid media by matrix-assist- ed laser desorption ionization-time of flight mass spec- trometry in the clinical laboratory. J Clin Microbiol 2013; 51(9): 2875-9. https://doi.org/10.1128/JCM.00819-13*
24. *
  Singh K, Kumari R, Tripathi R, Gupta S, Anupurba S. Detection of clinically important non tuberculous myco- bacteria (NTM) from pulmonary samples through one- step multiplex PCR assay. BMC Microbiol 2020; 20(1): 267. https://doi.org/10.1186/s12866-020-01952-y*
25. *
  Yu XL, Lu L, Chen GZ, Liu ZG, Lei H, Song YZ, et al. Identification and characterization of non-tuberculous mycobacteria isolated from tuberculosis suspects in Southern-central China. PLoS One 2014; 9(12): e114353. https://doi.org/10.1371/journal.pone.0114353*
26. *
  de Zwaan R, van Ingen J, van Soolingen D. Utility of rpoB gene sequencing for identification of nontuberculous mycobacteria in the Netherlands. J Clin Microbiol 2014; 52(7): 2544-51. https://doi.org/10.1128/JCM.00233-14*
27. *
  Tudó G, Monté MR, Vergara A, López A, Hurtado JC, Ferrer-Navarro M, et al. Implementation of MALDI-TOF MS technology for the identification of clinical isolates of Mycobacterium spp. in mycobacterial diagnosis. Eur J Clin Microbiol Infect Dis 2015; 34(8): 1527-32. https://doi. org/10.1007/s10096-015-2381-2*
28. *
  Maurya AK, Nag VL, Kant S, Kushwaha RA, Kumar M, Singh AK, et al. Prevalence of nontuberculous mycobacte- ria among extrapulmonary tuberculosis cases in tertiary care centers in Northern India. Biomed Res Int 2015; 2015: 465403. https://doi.org/10.1155/2015/465403*
29. *
  Mwikuma G, Kwenda G, Hang’ombe BM, Simulundu E, Kaile T, Nzala S, et al. Molecular identification of non-tu- berculous mycobacteria isolated from clinical specimens in Zambia. Ann Clin Microbiol Antimicrob 2015; 14: 1. https://doi.org/10.1186/s12941-014-0059-8*
30. *
  Wang HY, Kim H, Kim S, Kim DK, Cho SN, Lee H. Performance of a real-time PCR assay for the rapid identi- fication of Mycobacterium species. J Microbiol 2015; 53(1): 38-46. https://doi.org/10.1007/s12275-015- 4495-8*
31. *
  Mediavilla-Gradolph MC, De Toro-Peinado I, Bermúdez- Ruiz MP, García-Martínez Mde L, Ortega-Torres M, Montiel Quezel-Guerraz N, et al. Use of MALDI-TOF MS for iden- tification of nontuberculous mycobacterium species iso- lated from clinical specimens. Biomed Res Int 2015; 2015: 854078. https://doi.org/10.1155/2015/854078*
32. *
  Khosravi AD, Hashemzadeh M, Hashemi Shahraki A, Teimoori A. Differential identification of mycobacterial species using high-resolution melting analysis. Front Microbiol 2017; 8: 2045. https://doi.org/10.3389/ fmicb.2017.02045*
33. *
  Otchere ID, Asante-Poku A, Osei-Wusu S, Aboagye SY, Yeboah-Manu D. Isolation and characterization of nontu- berculous mycobacteria from patients with pulmonary tuberculosis in Ghana. Int J Mycobacteriol 2017; 6(1): 70-5. https://doi.org/10.4103/2212-5531.201895*
34. *
  Nasiri MJ, Dabiri H, Fooladi AAI, Amini S, Hamzehloo G, Feizabadi MM. High rates of nontuberculous mycobacte- ria isolation from patients with presumptive tuberculosis in Iran. New Microbes New Infect 2017; 21: 12-7. https:// doi.org/10.1016/j.nmni.2017.08.008*
35. *
  Verma AK, Sarin R, Arora VK, Kumar G, Arora J, Singh P, et al. Amplification of Hsp 65 gene and usage of restriction endonuclease for identification of non tuberculous rapid grower mycobacterium. Indian J Tuberc 2018; 65(1): 57-62. https://doi.org/10.1016/j.ijtb.2017.08.030*
36. *
  Tan Y, Su B, Shu W, Cai X, Kuang S, Kuang H, et al. Epidemiology of pulmonary disease due to nontubercu- lous mycobacteria in Southern China, 2013-2016. BMC Pulm Med 2018; 18(1): 168. https://doi.org/10.1186/ s12890-018-0728-z*
37. *
  Gharbi R, Mhenni B, Ben Fraj S, Mardassi H. Nontuberculous mycobacteria isolated from specimens of pulmonary tuberculosis suspects, Northern Tunisia: 2002- 2016. BMC Infect Dis 2019; 19(1): 819. https://doi. org/10.1186/s12879-019-4441-1*
38. *
  Furuuchi K, Morimoto K, Yoshiyama T, Tanaka Y, Fujiwara K, Okumura M, et al. Interrelational changes in the epide- miology and clinical features of nontuberculous mycobac- terial pulmonary disease and tuberculosis in a referral hospital in Japan. Respir Med 2019; 152: 74-80. https:// doi.org/10.1016/j.rmed.2019.05.001*
39. *
  Lin S, Wei S, Zhao Y, Lin J, Pang Y. Epidemiology of human pulmonary infection with nontuberculous mycobacteria in Southeast China: A prospective surveillance study. Infect Drug Resist 2019; 12: 3515-21. https://doi. org/10.2147/IDR.S223828*
40. *
  Pan X, Zhou Y, Li Z, Zhang J, Hong L, Shi Y, et al. Investigation of non-tuberculous mycobacteria in a prima- ry hospital from southeastern China. J Infect Dev Ctries 2019; 13(12): 1095-100. https://doi.org/10.3855/ jidc.11772*
41. *
  Karami-Zarandi M, Bahador A, Gizaw Feysia S, Kardan- Yamchi J, Hasan-Nejad M, Mosavari N, et al. Identification of non-tuberculosis mycobacteria by line probe assay and determination of drug resistance patterns of isolates in Iranian patients. Arch Razi Inst 2019; 74(4): 375-84.
  *
42. *
  Sharma M, Malhotra B, Tiwari J, Bhargava S. Profile of nontuberculous mycobacteria in patients suspected of tuberculosis and drug-resistant tuberculosis. J Lab Physicians 2020; 2(3): 203-11. https://doi. org/10.1055/s-0040-1721160*
43. *
  Sam AS, Ninan MM, Ranjani R, Devanga Raghupathi NK, Balaji V, Michael JS. Nontuberculous mycobacteria - clini- cal and laboratory diagnosis: Experiences from a TB endemic country. Future Sci OA 2020; 6(9): FSO612. https://doi.org/10.2144/fsoa-2020-0023*
44. *
  Singh AK, Maurya AK, Umrao J, Kant S, Kushwaha RA, Nag VL, et al. Role of GenoType(®) mycobacterium com- mon mycobacteria/additional species assay for rapid dif- ferentiation between Mycobacterium tuberculosis com- plex and different species of non-tuberculous mycobacte- ria. J Lab Physicians 2013; 5(2): 83-9. https://doi. org/10.4103/0974-2727.119847*
45. *
  Huang M, Tan Y, Zhang X, Wang Y, Su B, Xue Z, et al. Effect of mixed infections with Mycobacterium tuberculosis and nontuberculous mycobacteria on diagnosis of multi- drug-resistant tuberculosis: A retrospective multicentre study in China. Infect Drug Resist 2022; 15: 157-66. https://doi.org/10.2147/IDR.S341817*
46. *
  Smirnova T, Ustinova V, Andreevskaya S, Larionova E, Kiseleva E, Chernousova L, et al. Evaluation of a new assay for nontuberculous mycobacteria species identification in diagnostic material and cultures. Tuberculosis (Edinb) 2021; 130: 102124. https://doi.org/10.1016/j. tube.2021.102124*
47. *
  Lee YM, Kim MJ, Kim YJ. Increasing trend of nontubercu- lous mycobacteria isolation in a referral clinical laboratory in South Korea. Medicina (Kaunas) 2021; 57(7): 720. https://doi.org/10.3390/medicina57070720*
48. *
  Huang M, Lin Y, Chen X, Wu D. The value of gene chip detection of bronchoalveolar lavage fluid in the diagnosis of nontuberculous mycobacterial lung disease. Ann Palliat Med 2021; 10(6): 6438-45. https://doi.org/10.21037/ apm-21-1205*
49. *
  Asaoka M, Hagiwara E, Etori S, Higa K, Ikeda S, Sekine A, et al. Identification and characteristics of co-isolation of multiple nontuberculous mycobacteria. Intern Med 2021; 60(20): 3213-9. https://doi.org/10.2169/internalmedi- cine.5300-20*
50. *
  Kim MJ, Kim KM, Shin JI, Ha JH, Lee DH, Choi JG, et al. Identification of nontuberculous mycobacteria in patients with pulmonary diseases in Gyeongnam, Korea, using multiplex PCR and multigene sequence-based analysis. Can J Infect Dis Med Microbiol 2021; 2021: 8844306. https://doi.org/10.1155/2021/8844306*
51. *
  Huang JJ, Li YX, Zhao Y, Yang WH, Xiao M, Kudinha T, et al. Prevalence of nontuberculous mycobacteria in a tertia- ry hospital in Beijing, China, January 2013 to December 2018. BMC Microbiol 2020; 20(1): 158. https://doi. org/10.1186/s12866-020-01840-5*
52. *
  Almutairi T, Almohaya AM, Alqahtani A, Alkinani O, Alasmari F, Binkhamis K. Clinical characteristics and imag- ing features of patients with nontuberculous mycobacteria in a tertiary care center. J Clin Tuberc Other Mycobact Dis 2021; 26: 100294. https://doi.org/10.1016/j. jctube.2021.100294*
53. *
  Song J, Yoon S, In Y, Kim D, Lee H, Yong D, et al. Substantial improvement in nontuberculous mycobacterial identifica- tion using ASTA MicroIDSys matrix-assisted laser desorp- tion/ionization time-of-flight mass spectrometry with an upgraded database. Ann Lab Med 2022; 42(3): 358-62. https://doi.org/10.3343/alm.2022.42.3.358*
54. *
  Zhu Y, Hua W, Liu Z, Zhang M, Wang X, Wu B, et al. Identification and characterization of nontuberculous mycobacteria isolated from suspected pulmonary tuber- culosis patients in Eastern China from 2009 to 2019 using an identification array system. Braz J Infect Dis 2022; 26(2): 102346. https://doi.org/10.1016/j. bjid.2022.102346*
55. *
  Cassidy PM, Hedberg K, Saulson A, McNelly E, Winthrop KL. Non-tuberculous mycobacterial disease prevalencev and risk factors: A changing epidemiology. Clin Inf Dis 2009; 49(12): 124-9. https://doi.org/10.1086/648443*
56. *
  Stout JE, Koh WJ, Yew WW. Update on pulmonary disease due to non tuberculous mycobacteria. Int J Infect Dis 2016; 45: 123-34. https://doi.org/10.1016/j. ijid.2016.03.006*
57. *
  Zulu M, Monde N, Nkhoma P, Malama S, Munyeme M. Nontuberculous mycobacteria in humans, animals, and water in Zambia: A systematic review. Front Trop Dis 2021; 2: 679501. https://doi.org/10.3389/ fitd.2021.679501*
58. *
  Dahl VN, Mølhave M, Fløe A, van Ingen J, Schön T, Lillebaek T, et al. Global trends of pulmonary infections with nontuberculous mycobacteria: A systematic review. Int J Infect Dis 2022; 125: 120-31. https://doi. org/10.1016/j.ijid.2022.10.013*
59. *
  Bachar K, Shulimzon T, Segel MJ. Nontuberculous myco- bacteria infections of the pleura: A systematic review. Respir Med 2022; 205: 107036. https://doi.org/10.1016/j. rmed.2022.107036*
60. *
  Lee MR, Chang LY, Ko JC, Wang HC, Huang YW. Nontuberculous mycobacterial lung disease epidemiology in Taiwan: A systematic review. J Formos Med Assoc 2020; 119 (Suppl) 1: 4-12. https://doi.org/10.1016/j. jfma.2020.05.019*

